# The ST2/IL-33 Pathway in Adult and Paediatric Heart Disease and Transplantation

**DOI:** 10.3390/biomedicines11061676

**Published:** 2023-06-09

**Authors:** Giacomina Brunetti, Barbara Barile, Grazia Paola Nicchia, Francesco Onorati, Giovanni Battista Luciani, Antonella Galeone

**Affiliations:** 1 Department of Biosciences, Biotechnologies and Environment, University of Bari Aldo Moro, 70125 Bari, Italy; giacomina.brunetti@uniba.it (G.B.); barbara.barile@uniba.it (B.B.); graziapaola.nicchia@uniba.it (G.P.N.); 2 Department of Surgery, Dentistry, Pediatrics and Gynecology, Division of Cardiac Surgery, University of Verona, 37129 Verona, Italy; francesco.onorati@univr.it (F.O.); giovanni.luciani@univr.it (G.B.L.)

**Keywords:** ST2, cardiovascular disease, IL-33

## Abstract

ST2 is a member of interleukin 1 receptor family with soluble sST2 and transmembrane ST2L isoforms. The ligand of ST2 is IL-33, which determines the activation of numerous intracytoplasmic mediators following the binding with ST2L and IL-1RAcP, leading to nuclear signal and cardiovascular effect. Differently, sST2 is released in the blood and works as a decoy receptor, binding IL-33 and blocking IL-33/ST2L interaction. sST2 is mainly involved in maintaining homeostasis and/or alterations of different tissues, as counterbalance/activation of IL-33/ST2L axis is typically involved in the development of fibrosis, tissue damage, inflammation and remodeling. sST2 has been described in different clinical reports as a fundamental prognostic marker in patients with cardiovascular disease, as well as marker for the treatment monitoring of patients with heart failure; however, further studies are needed to better elucidate its role. In this review we reported the current knowledge about its role in coronary artery disease, heart failure, heart transplantation, heart valve disease, pulmonary arterial hypertension, and cardiovascular interventions.

## 1. Introduction

Growth stimulation expressed gene 2 (ST2) is a member of the interleukin-1 (IL-1) receptor superfamily also known as IL1RL-1, DER4, T1, and FIT-1 [1]. The scientific literature has been miscalling ST2 as “suppressor of tumorigenicity 2” (which is encoded by another gene), as growth stimulation expressed gene 2, and finally, it has been renamed by the initial discoverer as “serum stimulation-2” as it functions as a mediator of type 2 inflammatory response. ST2 was discovered by two different laboratories working on fibroblasts [2]. In 1989, Tominaga et al. cloned ST2 onto human chromosome 2q12.1, thus reporting that the protein showed an elevated sequence homology by comparing with immunoglobulin superfamily members [3], and the gene product was named ST2. Later in 2002, Weinberg et al. demonstrated that the translation and expression of sST2 were unusually overexpressed in cardiomyocytes with mechanical damage [4]. 

The *ST2* gene is located on human chromosome 2q12 [5,6]. The human *ST2* gene encodes four diverse isoforms arising from the alternative promotor splicing: soluble ST2 (sST2 also known as IL1RL1-a), membrane-bound receptor ST2 (ST2L also known as IL1RL1-b), ST2LV, and ST2V. Among the different isoforms, sST2 and ST2L represent the key transcriptional products. Different ST2 isoforms are known. Variant 1 (ST2L of about 556 amino acids) is the longest transcript and encodes for the full-length transmembrane isoform. The type I membrane receptor ST2L includes three extracellular IgG-like domains, an intracellular Toll/IL-1R (TIR) domain and a transmembrane domain [7]. ST2L is expressed by endothelial cells, colon cells, and different hematopoietic cells, (i.e., basophils, CD4^+^ T lymphocytes, macrophages, and eosinophils) [8,9]. Variant 2 (sST2, of about 328 amino acids) presents a distinctive C-terminus formed by five and nine amino acids in human and mice, respectively. As a soluble truncated form of ST2L, TIR and all transmembrane domains lack in sST2 [10]. sST2 can be secreted by myocardial cells, endothelial cells, alveolar epithelium and immune cells (i.e., T cells, macrophages, and mast cells), either constitutively or in response to stimulation [11,12]. In diseases, the sST2 source can differ accordingly, i.e., in myocardial injury, the molecule is secreted by cardiomyocytes and endothelial cells, of which hypertension-immune cells are the main producer. ST2LV (formed by 211 amino acids) and ST2V (formed by 259 amino acids) represent ST2L splicing variants due to exons difference. In detail, ST2LV, which is associated with the later stages of embryogenesis, does not include the ST2L transmembrane domains and is released by many tissues, such as the eyes, lungs, heart, and liver [13,14]. ST2V is characterized by an additional hydrophobic tail and the absence of an ST2L immunoglobulin-like domain [14]. ST2V can be found in the intestines and stomach [15]; however, ST2 was considered an orphan receptor, and its biological function unknown at that time until Schmitz et al. demonstrated that interleukin 33 (IL-33) represent the ST2 functional ligand [16]. 

The protein IL-33 is also known as C9ORF26, NF-HEV, DVS27, and IL-1F11. The gene for human and murine *IL-33* is located on chromosomes 9 (9p24.1) and 19 (19qc1), thus resulting in proteins with 270 (30 kDa) and 266 amino acids, respectively. IL-33 works as a classical and intracellular nuclear factor regulating the transcriptional activity, resulting in a dual-function cytokine [17]. IL-33 can work as a pro- or anti-inflammatory cytokine, according to the activated cell type, the microenvironment and the stimulus [18]. High IL-33 mRNA expression has been found in the brain, stomach, lung, spinal cord and skin by expression analysis of human cDNA libraries. Low levels of IL-33 were detected in the lymph tissue, spleen, kidney, pancreas and heart. IL-33 is mainly expressed by stromal cells, fibroblasts, endothelial cells, and smooth muscle cells [19,20].

**Figure 1:**
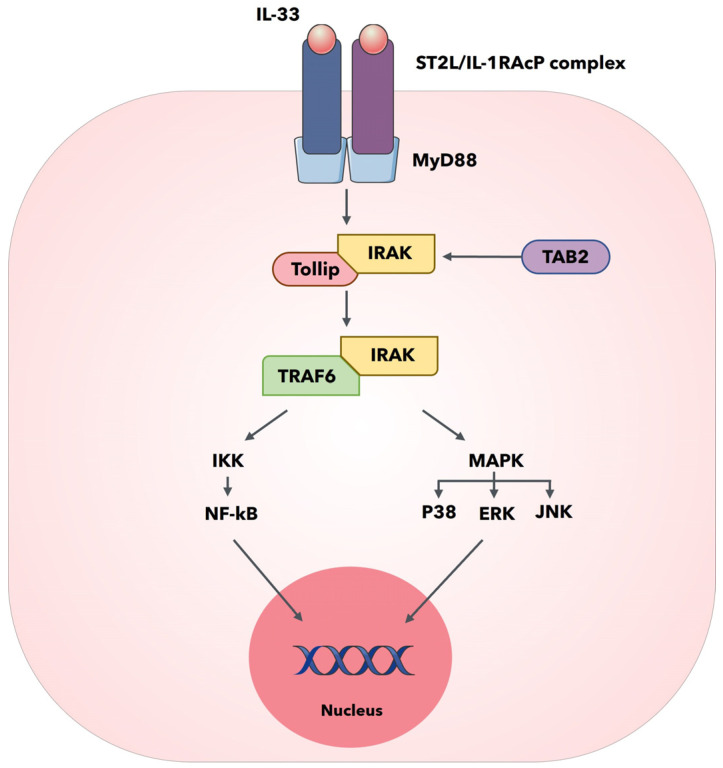
IL-33/ST2 signaling pathway activation. The graphical abstract was generated using Servier Medical 589 Art, provided by Servier, licensed under a Creative Commons Attribution 3.0 unported license.

IL-33 is mostly sited in the nucleus and contributes to maintaining cellular homeostasis through binding with chromatin and the regulation of RNA transcription [21]; however, IL-33 can be also secreted, and its key biological function is to bind the receptor complex composed of ST2L and IL-1RAcP, thus activating a signal transduction resulting in the involvement of myeloid differentiation primary response protein 88 (MyD88) by the activation of the TIR domain of IL-1RAcP (Figure 1) [22]. The induction of MyD88 N-terminal DD domain determines the recruitment of IL-1 receptor-associated kinase 4 (IRAK4) that begins to labor as a kinase to phosphorylate IRAK 1 (normally inactivated by the interaction with Toll-interacting protein (Tollip)), and also interacts with TNF receptor-associated factor 6 (TRAF6) [23]. Consequently, IRAK 1 reduces its affinity to Tollip for the phosphorylation and interaction with MyD88; TRAK1 and TRAF6 are further phosphorylated and consequently disconnects from MyD88. Next, TRAF6 ubiquitinates its amino acid, and binds and activates transforming growth factor β-activated kinase 1 (TAK 1) thanks to the mediation of TAB-1 (TAK 1 binding protein-1) and TAB-2 (TAK 1 binding protein-2) [24]. Activated TAK 1 phosphorylates mitogen-activated protein kinase (MAPK) and IκB kinase (IKK) complexes. Consequently, the IKK pathway determines Ca^2+^ mobilization with the consequent activation of the transcription factor nuclear factor-κB (NF-κB), whereas the MAPK pathway determines the activation of extracellular signal-regulated kinase (ERK), Jun N-terminal kinase (JNK) and p38, thus finally resulting with the production of different cytokines, such as TNF-α, IL-1, and IL-6 [25,26]. These soluble molecules are released in the extracellular microenvironment and develop a suitable immune response to shut down fibrosis, inflammation, and ventricular remodeling to safeguard the heart; however, sST2 also binds IL-33 as a decoy receptor, thus antagonizing the activation of the ST2L/IL-1RAcP receptor complex and altering the protecting effect of the IL-33/ST2 signaling pathway on heart because sST2 presents the same ST2L extracellular domain.

The inflammatory cytokines/chemokines released following the IL-33/ST2 interaction depends on the types of immune cells that express ST2L. 

### ST2 and the Cardiovascular System

The ST2/IL-33 pathway is primarily involved in inflammatory and autoimmune diseases, but it also has a main role in cardiovascular system and disease. ST2 has been shown to be upregulated in cultured rat cardiomyocytes subjected to mechanical strain [4], and its functional ligand IL-33 is also produced in response to stretch by cardiac fibroblast and has a cardioprotective role in the setting of myocyte stretch and injury [27]. In vitro, IL-33 blocks the phenylephrine-mediated cardiomyocyte hypertrophy and the actions of angiotensin-II; interestingly, IL-33 treatment seems to adjust hypertrophy and ventricular fibrosis in mice exposed to ventricular pressure overload, but only in wild-type mice, while the perturbation of the ST2 gene determines a progressive hypertrophy and myocardial fibrosis in animal models [27]. The biological effects of IL-33 are antagonized by sST2 as it binds to IL-33 and function as a decoy receptor to prevent IL-33 from binding to and signaling through ST2L [27]. While the lung has been shown to have the highest expression of sST2, other sources in the cardiovascular system include endothelial cells and cardiac myocytes, and the secretory capacity of these cells for sST2 is greatly enhanced by certain proinflammatory cytokines, such as tumour necrosis factor alpha (TNF-α) and IL-1β. Increased serum levels of sST2 can serve to limit the systemic biological effects of IL-33, resulting in inadequate cardioprotection from IL-33, with a heightened risk for adverse remodeling and ventricular dysfunction [27]. Following augmented cardiac loads (augmented pressure and biomechanical stress, and tension of the muscular structures) associated with acute myocardial infarction (AMI) or progressive heart failure (HF), cardiomyocytes, endothelial cells and cardiac fibroblasts increase the expression and release of both ST2 forms and IL-33 [4,28,29]. 

ST2L expressed on the surface of cardiomyocytes binds to IL-33 (secreted by cardiac fibroblasts upon biomechanical stress) and develops different cardioprotective effects. Different studies have reported that IL-33/ST2L binding is fundamental for the homeostasis, immune response, and the revitalization of injured heart muscle. This interaction decreases the apoptosis of cells subjected to ischemic and inflammatory processes, due to either infarction or the reduced circulation in the heart muscle. Furthermore, this interaction decreases cardiomyocyte hypertrophy and myocardial fibrosis, thus maintaining the ventricular function with consequent increase in patient survival [4,30,31]. The soluble receptor sST2 is concurrently secreted by endothelial cells and fibroblasts, thus working as a decoy receptor by competitively interacting with IL-33 and avoiding the interaction of IL-33 with ST2L and its cardioprotective effects on heart muscle [30,31]. Thus, the enhanced expression and secretion of sST2 increases cell apoptosis, fibrosis development, hypertrophy, remodeling of the heart muscle and heart failure progression. The ST2/IL-33 pathway is involved in many cardiovascular diseases, including ischemic and valvular heart disease, myocardial infarction (MI), heart failure (HF), myocarditis and cardiomyopathies. As a consequence of all the main discoveries in cardiovascular field of the last decades, the American College of Cardiology Foundation/Heart Association (ACCF/AHA) officially listed sST2 as a biomarker of myocardial fibrosis in 2013, whereas sST2 was utilized in the risk stratification of patients with HF [32]. Beetler et al. reported that age and sex differences in sera sST2 levels exist for patients with MI, myocarditis and cardiomyopathy, but were not observed in other cardiovascular disease, such as coronary artery disease and HF [33].

**Figure 2:**
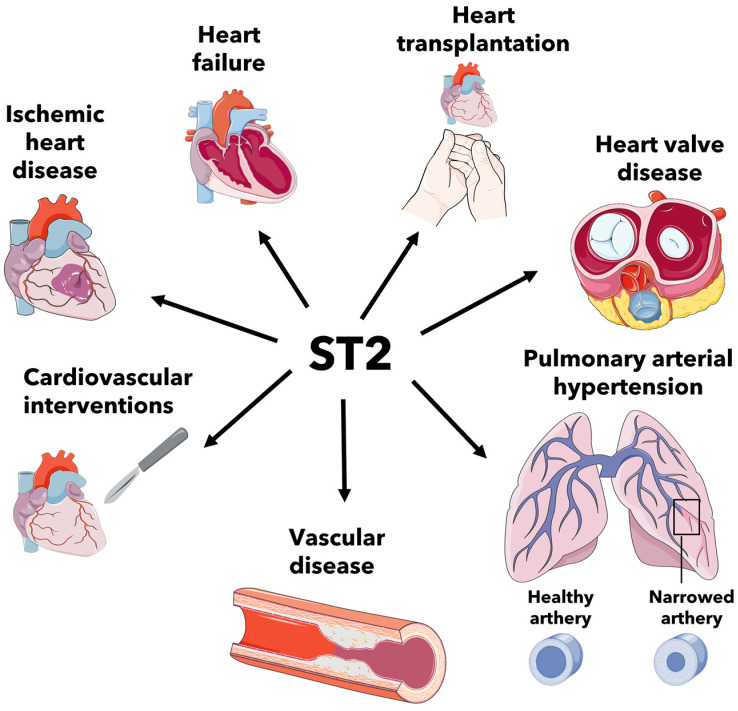
ST2 involvement in ischemic heart disease, heart failure, heart transplantation, heart valve disease, pulmonary arterial hypertension, vascular disease and cardiovascular interventions. The graphical abstract was generated using Servier Medical 589 Art, provided by Servier, licensed under a Creative Commons Attribution 3.0 unported license.

This review highlighted the role of ST2 in the different cardiovascular diseases (Figure 2).

## 2. ST2 Involvement in Cardiovascular Diseases

### 2.1. Ischemic Heart Disease 

Ischemic heart disease is the leading cause of death worldwide and represents a huge socioeconomic burden, thus the identification of risk stratification and prognostic biomarkers is an important matter of investigation. Myocardial ischemia is usually due to coronary atherosclerosis and occurs when coronary blood flow is reduced because of the occlusion of a coronary artery [34]. An irreversible injury due to severe and prolonged myocardial ischemia leads to MI with myocardial cell death and early revascularization may reduce the loss of contractile myocardial muscle mass, decrease the infarct size, and improve clinical outcome [35]; however, reperfusion not only rescue ischemic myocardium from infarction but also induces an additional irreversible injury known as myocardial ischemia–reperfusion injury [36]. Following MI, important modifications in the size and shape of the whole left ventricle occur that lead to left ventricular remodeling (LVR). The typical characteristics of postinfarction LV remodelling are represented by myocardial hypertrophy, infarct expansion, ventricular dilation, and cardiac fibrosis, arising from the inflammatory responses as well as the neuroendocrine activation [37,38]. ST2 induces the secretion of pro-inflammatory molecules from immune cells and could be involved in LVR and cardiac fibrosis. In an MI murine model, it has been demonstrated that IL-33 injection was associated with the alteration of ventricular remodelling and cardiac function [39]. Weinberg et coll. firstly described that the ST2 gene is markedly upregulated in cultured murine cardiomyocytes subjected to mechanical strain or after treatment with IL1β [4]. They additionally found that sST2 levels transiently increased after coronary artery ligation in mice; likewise, sST2 levels increase in the serum of patients 1 day after AMI and correlate positively with creatine kinase and negatively with left ventricular ejection fraction (LVEF) [4]. Later it has been shown that serum levels of sST2 predict mortality and heart failure in patients with AMI [40]. Jenkins et al. reported that sST2 is increased in more than half of patients with incident MI [41]. Elevated sST2 levels determine a high adverse prognosis with a great risk of death and heart failure over a long period of follow-up [41]. sST2 levels also are a significant predictor of cardiovascular death and HF independently of amino terminal B-type natriuretic peptide (NT-proBNP), and the combination of ST2 and NT-proBNP significantly improves risk stratification [42]. Serum sST2 levels are associated with LVEF early after AMI, but also with medium-term LV function; additionally, sST2 levels correlate to infarct magnitude and infarct remodeling over time [43]. Serum sST2 levels are also elevated in non-ST elevation acute coronary syndrome (NSTE-ACS) and predict early (30 days) and late (1 year) HF and mortality [44,45,46]. Kercheva et al. reported that in patients with primary MI with ST-segment elevation (STEMI) sST2 increase in sera was significantly related with LVR after 6 months [47]. Differently, Bière et al. showed that sST2 levels were associated with early LVR (<3 months) and late LVR (>3 months) [48]. Experimental data indicate that IL-33 prevents cardiomyocyte apoptosis and improves cardiac function and survival after myocardial infarction through ST2 signaling [30]. IL-33 protects cultured cardiomyocytes from hypoxia-induced apoptosis, suppresses caspase-3 activity, and increases expression of IAP family member proteins, and this cardioprotection is partially inhibited by sST2 [30]. IL-33 decreases both infarct and fibrosis volumes, and improves ventricular function and survival only in wild-type mice but not in ST2^−/−^ mice [30]. 

Further studies are warranted to evaluate if the ST2/IL-33 pathway could be a potential therapeutic target. The blocking of sST2, thus allowing the signaling of IL-33, could reduce myocardial reperfusion injury and prevent left ventricular remodeling in patients with acute MI.

### 2.2. Heart Failure 

Serum sST2 levels are significantly higher in patients with severe chronic HF and correlate with BNP levels; additionally, a change in sST2 levels over time is an independent predictor of subsequent mortality or the need for heart transplantation in these patients [49]. Increased serum levels of sST2 have also been reported in patients presenting with acute dyspnea in the emergency department setting, and concentrations of sST2 are higher among those with acute HF compared with those without HF; additionally, higher sST2 levels are associated with a higher risk of death at 1 year after presentation [50,51]. In patients with acute HF, increased sST2 values correlate with the severity of HF, LVEF, and NT-BNP; sST2 levels at presentation predict mortality at 1 year, and sST2 is equally predictive in patients with HF and preserved or impaired systolic function [51]. When both sST2 and natriuretic peptides are elevated, the highest rates of death are observed, but in the presence of low sST2 levels, natriuretic peptides do not predict mortality [51]. Acute destabilized HF sST2 levels in patients are predictive of 1-year mortality [52], and a change in sST2 concentrations during acute HF treatment is predictive of 90-day mortality and is independent of BNP or NT-proBNP levels [53]. Experimental studies suggest that the overloaded myocardium is not the major source of sST2 in HF. Indeed, endothelial cells possess a functional ST2 secretory system in vitro, suggesting that the vascular endothelium could be responsible for sST2 production in vivo [54]. Human cardiac and vascular cells show important differences in the distribution patterns of ST2 isoform mRNA expression and produce different amounts of sST2 protein [55]. Both cardiac macrovascular (aortic and coronary artery) and microvascular endothelial cells express specific mRNA for both ST2 isoforms and are a source for sST2 protein, whereas cardiac myocytes, fibroblasts and vascular SMC express only minor amounts of ST2 mRNA and do not secrete detectable amounts of sST2 antigen [55]. Human cardiac fibroblasts, myocytes and coronary artery SMC did not respond to treatment with IL-33. In fact, recombinant human IL-33 did not induce NF-κB p50 and p65 subunit nuclear translocation or increase IL-6, IL-8, and monocyte chemoattractant protein (MCP-1) levels in these cells [55]. Indeed, endothelial cells are the source of sST2 and the target for IL-33 in the cardiovascular system. IL-33 is expressed in the nucleus of human adult cardiac fibroblasts and myocytes, and it is released following necrosis of these cells. In vitro proinflammatory cytokines, such as TNF-α, IFN-γ and IL-1β increase IL-33 expression in these cells, and in myocardial tissue from patients undergoing heart transplantation, IL-33 mRNA levels correlated with TNF-α and IFN-γ mRNA expression [55]. The lungs are also a relevant source of sST2 in heart failure. In an experimental model of ischemic heart failure sST2, ST2L, and IL-33 were measured in lungs, heart, kidney and liver by quantifying mRNA and protein expression in tissue samples obtained at different times. sST2 increased significantly from the first week in both lungs and myocardium, whereas ST2L/IL-33 response decreased in lungs and increased myocardium. No changes were observed in liver and kidneys. In vivo, sST2 measured in samples of bronchial aspirate and serum obtained from patients treated with invasive respiratory support showed that sST2 levels in lung aspirates were substantially higher in patients with cardiogenic pulmonary edema compared to patients with bronchopneumonia or neurological disorders [56].

The effects of pharmacological treatment of HF on sST2 levels have been evaluated in patients enrolled in the PARADIGM-HF trial (Prospective Comparison of ARNI With ACEI to Determine Impact on Global Mortality and Morbidity in Heart Failure), along with the relationships between sST2 and outcomes, and the prognostic utility of sST2. sST2 levels were compared between treatment groups (sacubitril/valsartan versus enalapril) at baseline, 1 month and 8 month postrandomization. Relationships between baseline sST2 and HF hospitalization, cardiovascular death, and combined HF hospitalization and cardiovascular death were also assessed. Treatment with sacubitril/valsartan resulted in greater reductions and less increases in sST2 levels compared to enalapril. Changes in sST2 levels from baseline to 1 month were independently associated with outcomes. sST2 increase at 1 month was associated with worse subsequent outcomes, while sST2 decrease was associated with better outcomes [57].

In patients with end-stage HF, left ventricular assist device (LVAD) support results in a significant drop in sST2 levels with normalization within 3 months postimplantation, suggesting that LVAD support leads to reduction of fibrosis and inflammation. Cardiogenic shock and increased C-reactive protein levels are associated with higher sST2 levels in these patients [58]. 

Serum sST2 levels are associated with adverse events and also have strong prognostic value in patients with pediatric dilated cardiomyopathy; serial measurements of sST2 could help in managing these patients for monitoring outcomes of treatment [59]. Another study failed to demonstrate the diagnostic utility of sST2 in pediatric heart failure due to cardiomyopathy and congenital heart disease [60]. On the contrary, in adults with complex congenital heart disease, the most important predictors of acute heart/Fontan failure were sST2, NYHA class III/IV, NT-proBNP and γGT levels, while all-cause mortality was best predicted by sST2 levels [61]. Similarly, Geenen and coll. reported a significant association between sST2 and a primary composite endpoint of all-cause mortality, HF, hospitalization, arrhythmia, thromboembolic events or cardiac interventions in 602 patients with adult congenital heart disease [62]. 

sST2 are also elevated in patients with fulminant myocarditis and show a marked dynamic change from disease onset to resolution. The plasma sST2 level was able to differentiate fulminant myocarditis from nonfulminant myocarditis acute HF better than NT-pro-BNP or cardiac troponin I [63]. Plasma sST2 levels in patients with inflammatory cardiomyopathy reflect the degree of LV functional impairment at hospital admission and predict functional NYHA class at mid-term follow-up. Hence, ST2 may be helpful in the evaluation of disease severity and in the prediction of the clinical status in these patients [64]. Increased ST2 levels were found in sera of patients affected by Duchenne muscular dystrophy (DMD), a rare and fatal X-linked disorder characterized by the lack of dystrophin, a key sarcolemma muscle protein, in which cardiac failure represents a significant cause of death [65]. Of note in this study is that differences were seen in other biomarkers levels of HF as BNP and galectin-3 between patients with DMD and healthy volunteers [65].

ST2 is included in the American College of Cardiology/American Heart Association guidelines for additive risk stratification of patients with acute and chronic HF [32], and ST2 immunoassays are approved for clinical use. Serial evaluation of sST2 levels could be useful for clinicians for diagnosis, monitoring of therapy and prevention of rehospitalization in patients with HF. 

### 2.3. Heart Transplantataion

Previous studies demonstrated the involvement of ST2/IL-33 in the context of acute rejection after heart transplantation, showing a dynamic behavior of sST2 in association with rejections in heart recipients [66,67]. Serum sST2 levels rise significantly in the context of acute rejection, and there is a graded increase in rejection rates with rising concentrations of sST2; sST2 levels also show a significant linear association with severity of acute rejection and significantly decline after successful rejection therapy [66]. Soluble ST2 concentrations correlate positively with NT-pro-BNP levels, right atrial pressure, serum creatinine and C-reactive protein concentrations, and total white blood cell count, whereas they correlate inversely with time after transplantation, blood levels of immunosuppressive agents and LVEF [66]. Elevated sST2 levels in heart recipients are associated with cellular rejection and predict long-term mortality following heart transplant [67]. Other authors observed a statistical difference in the sST2 level according to the grades of the allograft rejection; however, after correction for confounding factors of the time after heart transplant, the statistical difference was not found [68].

Circulating sST2 levels are also elevated during acute rejection in pediatric heart recipients, and there is evidence for increased ST2 expression in pediatric heart biopsies during allograft rejection, suggesting the graft as a source of serum sST2 [69].

Heart recipients with higher postoperative sST2 levels (≥35 ng/mL) measured 1 year after transplant had a significantly higher incidence of antibody-mediated rejection; however no association was found between post-HTx sST2 level status and other post-HTx outcomes including survival [70].

Changes in sST2 levels could be explained by the myocardial injury caused by the acute rejection episode, similar to the increase in sST2 observed in AMI or acutely decompensated HF. ST2/IL-33 signaling is also involved in immune responses mediated by Th2 and promotes immune deviation toward Th2 function, which is associated with the induction of tolerance after transplantation [71]; therefore, IL33/ST2 signaling could have an additional cardioprotective role in the transplanted heart by facilitating immune tolerance and decreasing the risk of rejection. Thus, dysregulation of sST2 expression could be associated with a higher risk of rejection as sST2 acts as a decoy receptor and inhibits the IL33/ST2L binding. Experimental studies show that IL-33 prolongs murine cardiac allograft survival through the induction of Th2 immune responses [72,73]. The administration of IL-33 after a fully allogenic mouse heart transplantation results in significant graft prolongation associated with increased Th2-type responses and decreased systemic CD8+ IFN-γ+ cells [73]. Additionally, IL-33 increases CD11b+ cells that exhibit potent T cell suppressive function and causes an ST2-dependent expansion of suppressive CD4+ Foxp3+ regulatory T cells, including an ST2L+ population CD4+ Foxp3+ [73].

Allograft or recipient ST2 deficiency oppositely affected cardiac allograft vasculopathy, suggesting that interrupting IL-33/ST2 signaling locally or systematically after heart transplantation leads to different outcome. In a murine model of chronic cardiac allograft rejection, ST2 deficiency was significantly associated with allograft vascular occlusion and fibrosis, and increased macrophages and CD3+ T cells infiltration in allografts. In contrast, allografts ST2 deficiency resulted in decreased infiltration of macrophages, CD3+ T cells and CD20+ B cells, and reduced vascular occlusion and fibrosis of allografts [74]. 

A recent study explored the role of sST2 serum levels in heart donors and allograft function following heart transplantation for the first time [75]. The authors demonstrated that brain-dead organ donors have considerably high levels of sST2. The higher levels were observed in older donors and in donors with reduced LVEF, or have need for inotropic support and after-cardiac-arrest resuscitation. Additionally, elevated sST2 serum levels in donors were associated with allograft dysfunction in heart recipients [75]. Assessment of biomarkers is of paramount importance in potential heart donors as it may provide important predictive information and allows for risk stratification, especially in marginal donors, who are increasingly accepted due to the for scarcity of heart donors [76].

Soluble ST2 appears as a promising biomarker both in the assessment of heart donors and in the management of heart recipients; routine serum sST2 quantification in recipients after heart transplant, potentially combined with other biomarkers, could help in the non-invasive diagnosis of allograft rejection.

### 2.4. Heart Valve Disease

Some studies focused on the involvement of ST2 in heart valve disease, including aortic stenosis (AS) and mitral regurgitation (MR). Sawada and coll. investigated the expression and cellular localization of ST2 and IL-33 in human nonrheumatic stenotic aortic valves and found that ST2 expression was markedly upregulated in stenotic valves compared to nonstenotic valves; the molecular weight of ST2 was 50 kDa but not 100 kDa, suggesting that upregulated ST2 in stenotic valves was sST2 [77]. IL-33 was expressed in both stenotic and nonstenotic valves, but the expression levels were not significantly different between stenotic and nonstenotic valves [77]. Immunohistochemical analysis showed that sST2 and IL-33 were mainly colocalized with CD68 positive macrophages in stenotic valves, suggesting that sST2 may participate in the pathophysiology of calcific aortic valve disease via the inhibition of IL-33 in macrophages [77].

Serum levels of sST2 are significantly elevated in patients with AS compared to control patients, while RNA levels of membrane-anchored ST2L from left ventricle biopsies are significantly decreased in patients with AS compared those of to control patients [54]. Despite experimental evidence of load induction of ST2 mRNA in neonatal rat cardiac myocytes [4], the source of increased serum sST2 levels in pressure overload hypertrophy appears to be the vascular endothelium and not the myocardium [54].

Another study aimed to compare ST2 concentrations in patients with left ventricular systolic dysfunction from nonvalvular causes and patients with severe AS and found that sST2 levels did not differ significantly between patients with HF and patients with AS and normal EF (EF ≥ 50%); however, in AS patients with low EF (EF < 50%), ST2 concentrations were significantly higher than that of the HF group [78]. Lancelloti and coll. showed that in patients with AS and preserved LVEF fraction, both BNP and sST2 are associated with NYHA class, but sST2 (>23 ng/mL, AUC = 0.68, *p* < 0.01) is more accurate in identifying patients who will develop heart failure symptoms during follow-up than BNP [79]. In AS patients, sST2 is independently related to the left atrial index and aortic valve area; at multivariable analysis, peak aortic jet velocity and sST2 level were independent predictors of cardiovascular events [79]. In patients with severe AS, replacement myocardial fibrosis measured by cardiac magnetic resonance was associated with higher circulating sST2 levels, left ventricular hypertrophy and dilation, and lower LVEF. Of note, sST2 levels were also associated with the pattern of replacement myocardial fibrosis, being higher in midwall than in subendocardial fibrosis. Multivariate analyses showed that LV ejection fraction and sST2 levels were associated with myocardial fibrosis, and the presence of myocardial fibrosis was predicted as sST2 ≥ 28.2 ng/mL [80]. 

An in vitro and in vivo study showed that sST2 promotes oxidative stress and inflammation contributing to cardiac damage in patients with AS. In vitro sST2 downregulated mitofusin-1 (MFN-1), a protein involved in mitochondrial fusion in human cardiac fibroblasts, increased nitrotyrosine, protein oxidation and peroxide production, and enhanced the secretion of pro-inflammatory cytokines IL-6, IL-1β and monocyte chemoattractant protein-1 (CCL-2). Oxidative stress and inflammation induced by sST2 in cardiac fibroblasts was reduced by blocking NFκB or mitochondrial reactive oxygen species. The blocking of NFκB or mitochondrial reactive oxygen species also restored MFN-1 levels. In myocardial biopsies from AS patients, sST2 inversely correlated with MFN-1 levels and was positively associated with IL-6 and CCL-2 [81]. 

The ST2/IL-33 system is also activated in patients with chronic degenerative MR [82]. Preoperative ST2 activation, evidenced by the higher serum sST2 levels, is associated with higher LVEF and lower levels of left ventricular end-diastolic diameter after mitral valve repair, thus confirming the cardioprotective role of the ST2/IL-33 system [82].

Experimental studies suggest that IL-33/ST2 system may be involved in the development of myxomatous mitral valve disease by enhancing extracellular matrix remodeling. In patients undergoing mitral valve replacement for severe MR due to myxomatous degeneration, immunohistochemistry of IL-33 and ST2 are expressed in both valve interstitial cells (VICs) and valve endothelial cells (VECs) of the resected leaflets [83]. The levels of IL-33 were positively correlated with proteoglycans, matrix metalloproteinases and their tissue inhibitors, and inflammatory and fibrotic markers. In vitro stimulation of single cell cultures of VICs and VECs with recombinant human IL-33 induced the expression of activated VIC markers, endothelial-mesenchymal transition of VECs, proteoglycan synthesis, inflammatory molecules and extracellular matrix turnover [83].

Few studies explored the role of the IL33/ST2 pathway in the context of heart valve disease, suggesting that it could be involved in the pathogenesis of aortic and mitral valve disease; however, the high levels of sST2 observed in heart valve disease could also be related to heart failure symptoms developed by these patients. 

### 2.5. Pulmonary Arterial Hypertension

Pulmonary arterial hypertension (PAH) is classified as primary (idiopathic) or secondary to other pathologies. The pathogenesis of PAH is characterized by three major processes, including vasoconstriction, vascular remodeling with smooth-muscle cell and endothelial cell proliferation and microthrombotic events [84]. Evidence from animal models and studies in patients with PAH suggest that inflammation also contributes to the development of PAH [85]. It has been proposed that the IL-33/ST2 ligand interaction may be involved in the development of PAH. Previous studies showed that IL-33, the circulating ligand of sST2, may play a role in the vascular remodeling of the pulmonary endothelium in patients with idiopathic PAH (IPAH) [86]. In these patients nuclear IL-33 is markedly diminished in endothelial cells, and this correlates with reduced IL-33 mRNA expression in their lung. In contrast, serum levels of IL-33 are unchanged in patients with IPAH, while sST2 expression is enhanced in the serum of these patients [86]. The knocking down of IL-33 in human endothelial cells (ECs) using siRNA is associated with selective modulation of inflammatory genes involved in vascular remodeling, including IL-6. Additionally, IL-33 knock-down significantly increased sST2 release from endothelial cells [86].

Levels of sST2 are elevated in patients with PAH of different etiologies compared to healthy controls and have been correlated with right ventricular dysfunction and hemodynamic impairment in patients in these patients [87,88,89]. In patients with IPAH a significant association was also found between sST2 and clinical worsening [90]. Another study found a significant association between sST2 in patients with various types of PAH except due to left heart disease and found higher levels of sST2 in patients who were hospitalized for heart failure or died during follow-up [91].

A prospective study on a large cohort of patients with PAH of various etiologies investigated the prognostic role of sST2 and demonstrated a significant association between sST2 and the transplant-free survival [92]. In this study higher sST2 were associated with worse right ventricular dysfunction and higher mean pulmonary and right atrial pressures. sST2 was significantly associated with death, lung transplantation or heart failure; however, after adjustment for NT-proBNP, associations did not reach statistical significance [92].

Soluble ST2 is also a useful biomarker in pediatric populations with PAH. Elevated ST2 are associated with unfavorable pulmonary hemodynamics and functional measures, clinical worsening, and significantly improved prediction of clinical worsening in pediatric populations with PAH [93].

The lung has the highest expression of sST2, thus sST2 has been suggested as a promising biomarker for the prediction of severity and outcome in patients with PAH. Indeed, it could be more sensitive and superior than NT-proBNP as a biomarker for PAH because the latter is secreted mainly from ventricular tissue.

### 2.6. Vascular Disease 

ST2 and IL-33 are expressed in endothelial cells and may play an important role in vascular biology and the development of hypertension and atherosclerosis. Serum sST2 levels are increased in patients with hypertension [94], and higher sST2 concentrations in subjects free of hypertension are associated with incident hypertension over 3 years of follow-up [95]. Plasma ST2 levels are increased in hypertensive patients with left ventricular hypertrophy (LVH) and are highest in those with concentric hypertrophy [96]. Patients with hypertensive HF have higher plasma ST2 concentrations compared to patients with hypertensive LVH and to those with hypertension without LVH [97].

Experimental studies indicate that IL-33 can reduce atherosclerosis development in ApoE^-/-^ mice on a high-fat diet by increasing serum levels of IL-5 and the production of antioxidized low-density lipoprotein antibodies, while mice treated with sST2 develop significantly larger atherosclerotic plaques [98]. Conversely, clinical studies failed to provide association between sST2 levels and advanced atherosclerotic disease [99]. In patients with significant carotid artery stenosis, no differences were observed in sST2 levels between asymptomatic and symptomatic patients, and there was no association between sST2 levels and the histopathological features of the plaque, including size of the lipid core, degree of calcification, number of macrophages or SMCs, amount of collagen and number of microvessels [99]. Additionally, sST2 plasma levels had no predictive value for future cardiovascular events in patients with significant carotid artery stenosis [99].

Recent studies showed that sST2 might be a useful biomarker for acute aortic dissection in the emergency room, as it could discriminate acute aortic dissection from other causes of sudden-onset severe chest pain. sST2 levels are higher in patients with acute aortic dissection than in those with either acute MI within 24 h of symptom onset or in patients with pulmonary embolism [100]. Among patients with suspected aortic dissection in the emergency department, sST2 showed superior overall diagnostic performance to D-dimer or cardiac troponin I [100]. 

Previous studies investigated the role of ST2 in vascular disease with controversial results. Additional studies are required to better elucidate the role of these biomarkers in the diagnosis and prognosis of patients with vascular disease.

### 2.7. Cardiovascular Interventions

Serum sST2 levels significantly increase after cardiovascular interventions, including cardiac surgery with cardio-pulmonary by-pass (CPB), percutaneous coronary intervention (PCI) and peripheral vascular surgery. Cardiac surgery with CPB leads to a systemic inflammatory response syndrome [101,102], and both surgical stress and CPB represent an acute injury that leads to the prompt activation of the ST2 pathway and the secretion of high amounts of sST2. Cardiac surgery with CPB induces a rapid and transitory elevation of sST2 with a peak on the first postoperative day [82,103].

sST2 is also emerging as a prognostic biomarker in adult patients undergoing cardiac surgery. Preoperative sST2 levels are associated with postoperative acute kidney injury (AKI) risk and could be useful in identifying patients at higher risk of developing AKI after cardiac surgery [104]. In patients undergoing coronary artery bypass grafting, those with the highest pre- and postoperative levels of sST2 experienced significantly greater odds of in-hospital death compared to patients with the lowest sST2 values [105]. The addition of both postoperative and pre-to-postoperative sST2 biomarker significantly improved ability to predict in-hospital mortality after CABG surgery, compared to using the EuroSCORE II mortality model alone [105]. In two prospective cohorts of over 1800 patients who underwent cardiac surgery, higher postoperative sST2 levels were significantly associated with cardiovascular event or mortality. These associations were not significantly modified by preoperative congestive heart failure or acute kidney injury [106]. 

In patients receiving veno-arterial extracorporeal membrane oxygenation (V-A ECMO) support for postcardiotomy cardiogenic shock, sST2 levels at 24 h after V-A ECMO initiation were associated with continuous renal replacement therapy (CRRT) and could predict CRRT use in these patients [107].

Parker and coll. investigated the relationship between pre- and postoperative ST2 biomarker levels and the risk of readmission within 1 year after congenital heart surgery in 145 pediatric patients aged < 18 years [108]. The authors found that elevated serum levels of ST2 measured preoperatively and postoperatively were associated with increased risk of unplanned readmission within 1 year after pediatric congenital heart surgery [108]. 

sST2 levels are significantly elevated in patients after PCI and peripheral vascular surgery [109]. In a study on 1607 patients with STEMI undergoing PCI, preprocedural sST2 levels were higher in the no-or-slow-flow group and were independently associated with postprocedural no-or-slow flow [110]. In 205 patients with NSTE-ACS undergoing PCI, the sST2 concentration on admission was correlated with the degree of coronary artery stenosis and was an independent predictor of major adverse cardiac and cerebrovascular events and no-reflow phenomenon after PCI [111].

sST2 is useful for risk stratification and prediction of mortality in patients undergoing transcatheter aortic valve implantation (TAVI). A significant increase in sST2 levels has been observed after successful TAVI, probably due to a periprocedural myocardial injury associated with a transient LV dysfunction [112]. 

A study enrolling 461 patients undergoing TAVI showed that patients with sST2 > 29 ng/mL had an increased 30-day and 1-year mortality [113]. In multivariate regression analysis, independent predictors of mortality were sST2, logistic EuroSCORE, chronic renal failure, and LVEF and sST2 was independently associated with adverse outcomes after TAVI but was not superior to NT-proBNP or surgical risk scores for the prediction of postprocedural outcomes [113]. In a prospective study on 74 patients undergoing TAVI for severe AS, sST2 was higher in these patients compared to controls and significantly predicted survival and major adverse cardiovascular events, and adding sST2 to the established STS score improved prediction of two-year mortality in this cohort [114]. sST2 levels were associated with 1-year mortality after TAVI and might help to identify patients at high risk for death in whom conservative treatment should be considered [115]. 

A study conducted on 210 patients with MR undergoing percutaneous mitral valve repair using the MitraClip, showed that ST2 plasma levels were associated with successful MR reduction these patients [116]. 

All this evidence supports the idea that sST2 can predict morbidity and mortality after different cardiovascular interventions. Interestingly, both invasive and less invasive procedures are associated with activation of the IL33/ST2 pathway.

## 3. Conclusions

The ST2/IL-33 represents one of the most complex pathways as it is involved in different pathophysiological conditions, and also in cardiovascular diseases. The detailed knowledge of this pathway is of paramount importance because ST2 is helpful to diagnose, manage and monitor the treatment of many cardiac pathologies in which inflammation, apoptosis and fibrosis represent the main features. 

Until now, ST2 satisfies all criteria to represent a valid therapeutic and prognostic marker in patients with HF; additional studies are needed to identify if sST2 could also be a potential therapeutical target in this clinical condition.
